# Effect of Treating Streptozotocin-Induced Diabetic Rats With Sorbinil, Myo-Inositol or Aminoguanidine on Endoneurial Blood Flow, Motor Nerve Conduction Velocity and Vascular Function of Epineurial Arterioles of the Sciatic Nerve

**DOI:** 10.1080/15604280212525

**Published:** 2002

**Authors:** Lawrence J. Coppey, Jill S. Gellett, Eric P. Davidson, Joyce A. Dunlap, Mark A. Yorek

**Affiliations:** 1 Veterans Affairs Medical Center Diabetes Endocrinology Research Center and Department of Internal Medicine University of Iowa Iowa City IA 52246 USA; 2 3 E 17 Veterans Affairs Medical Center Diabetes Endocrinology Research Center and Department of Internal Medicine University of Iowa Iowa City IA 52246 USA

## Abstract

Previously we have demonstrated that diabetes causes impairment in vascular function of epineurial vessels, which precedes the slowing of motor nerve conduction velocity. Treatment of diabetic rats with aldose reductase inhibitors, aminoguanidine or myo-inositol supplementation have been shown to improve motor nerve conduction velocity and/or decreased endoneurial blood flow. However, the effect these treatments have on vascular reactivity of epineurial vessels of the sciatic nerve is unknown. In these studies we examined the effect of treating streptozotocininduced rats with sorbinil, aminoguanidine or myo-inositol on motor nerve conduction velocity, endoneurial blood flow and endothelium dependent vascular relaxation of arterioles that provide circulation to the region of the sciatic nerve. Treating diabetic rats with sorbinil, aminoguanidine or myo-inositol improved the reduction of endoneurial blood flow and motor nerve conduction velocity. However, only sorbinil treatment significantly improved the diabetes-induced impairment of acetylcholinemediated vasodilation of epineurial vessels of the sciatic nerve. All three treatments were efficacious in preventing the appropriate metabolic derangements associated with either activation of the polyol pathway or increased nonenzymatic glycation. In addition, sorbinil was shown to prevent the diabetes-induced decrease in lens glutathione level. However, other markers of oxidative stress were not vividly improved by these treatments. These studies suggest that sorbinil treatment may be more effective in preventing neural dysfunction in diabetes than either aminoguanidine or myoinositol.


*Int. Jnl. Experimental Diab. Res.*, 3:21-36, 2002
© 2002 Taylor and Francis
1560-4284/02 $12.00 + .00

### Effect of Treating Streptozotocin-induced Diabetic Rats With Sorbinil, Myo-inositol or Aminoguanidine on Endoneurial Blood Flow, Motor Nerve Conduction Velocity and Vascular Function of Epineurial Arterioles of the Sciatic Nerve

LAWRENCE J. COPPEY, JILL S. GELLETT, ERIC P. DAVIDSON, JOYCE A. DUNLAP, AND MARK A. YOREK*

Veterans Affairs Medical Center, Diabetes Endocrinology Research Center and Department of Internal Medicine, University of Iowa, Iowa City, IA, 52246, USA

*Received: July 2, 2001; In final form:October 29, 2001*

**Previously we have demonstrated that diabetes causes impairment in vascular function of epineurial vessels, which precedes the slowing of motor nerve conduction velocity. Treatment of diabetic rats with aldose reductase inhibitors, aminoguanidine or myo-inositol supplementation have been shown to improve motor nerve conduction velocity and/or decreased endoneurial blood flow. However, the effect these treatments have on vascular reactivity of epineurial vessels of the sciatic nerve is unknown. In these studies we examined the effect of treating streptozotocin-induced rats with sorbinil, aminoguanidine or myo-inositol on motor nerve conduction velocity, endoneurial blood flow and endothelium-dependent vascular relaxation of arterioles that provide circulation to the region of the sciatic nerve. Treating diabetic rats with sorbinil, aminoguanidine or myo-inositol improved the reduction of endoneurial blood flow and motor nerve conduction velocity. However, only sorbinil treatment significantly improved the diabetes-induced impairment of acetylcholine-mediated vasodilation of epineurial vessels of the sciatic nerve. All three treatments were efficacious in preventing the appropriate metabolic derangements associated with either activation of the polyol pathway or increased nonenzymatic glycation. In addition, sorbinil was shown to prevent the diabetes-induced decrease in lens glutathione level. However, other mark-**

*Corresponding author: 3 E 17 Veterans Affairs Medical Center, Iowa City, IA 52246; tel: (319) 338-0581 ext. 7629; fax: (319) 339-7025; e-mail: myorek@icva.gov

**ers of oxidative stress were not vividly improved by these treatments. These studies suggest that sorbinil treatment may be more effective in preventing neural dysfunction in diabetes than either aminoguanidine or myo-inositol.**

*Key words*: diabetes, diabetic neuropathy, endothelium, vascular reactivity, aldose reductase inhibitor, aminoguanidine

### INTRODUCTION

Diabetic neuropathy is a multifactorial problem likely due to the poor control of hyperglycemia. Two of these perturbations are the activation of the polyol pathway by which glucose is metabolized to sorbitol via aldose reductase contributing to an alteration in the redox balance and a decrease in myo-inositol uptake and content and an increase in non-enzymatic glycation leading to the abnormal glycation of proteins [1,2]. In animal models of diabetes prevention of these defects by treatment with aldose reductase inhibitors, aminoguanidine or supplementation with myo-inositol has been shown to improve peripheral neuropathy.

Studies from numerous laboratories have shown that treating diabetic rats with an aldose reductase inhibitor improves nerve function as well as endoneurial blood flow [3-12]. This has led to wide speculation regarding the potential benefit of aldose reductase inhibitor treatment of diabetic complications [10-16]. However, clinical trials using aldose reductase inhibitors for treatment of diabetic neuropathy have been disappointing [17]. Treatment of diabetic rats with dietary myo-inositol has also been shown to improve nerve function [18-21]. This led to speculation 15 years ago that a common mechanism might induce the diverse complications of diabetes [22]. However, more recent studies have indicated that diabetic neuropathy is likely due to multiple mechanisms involving hyperglycemia and decreased insulin and C-peptide levels that effect both nerve and vascular tissue [17].

Another mechanism thought to contribute to diabetic complications is non-enzymatic glycation of proteins. Treatment of diabetic rats with aminoguanidine, an inhibitor of the formation of advanced glycosylation end products [23], ameliorates the slowing of motor nerve conduction velocity and reduction of endoneurial blood flow [24-28]. This suggests that the formation of advanced glycation end products in the nerve or vascular tissue perturbs neural function [29-32].

Recently, Cameron et al. reported that diabetes causes a reduction in endoneurial blood flow and that endoneurial hypoxia is an important factor underlying nerve conduction deficits early in the development of diabetic neuropathy [33]. In their studies, they showed that nerve blood flow was reduced as early as 1 week after diabetes induction. Moreover, Wright and Nukada, and our laboratory have shown that reductions in nerve blood flow preceded the decrease in nerve conduction velocities [34,35]. In addition, we have shown that endothelial-dependent vasodilation of arterioles that provide circulation to the region of the sciatic nerve is impaired by diabetes and the reduction in vascular function also precedes slowing of motor nerve conduction velocity [35,36]. These studies suggest that vascular dysfunction is an important factor underlying nerve conduction deficits and reduced endoneurial blood flow early in the development of diabetic neuropathy. This is supported by earlier studies that demonstrated diabetes-induced impairment in vasoreactivity in the sciatic nerve is due to reduced nitric oxide mediated endothelium-dependent relaxation [37,38].

In spite of the large number of studies regarding the efficacy of treatment of diabetic rats with aldose reductase inhibitors, aminoguanidine or myo-inositol on neural activity little information is available on the effects of these treatments on vascular reactivity. In studies using aldose reductase inhibitors, treatment with tolrestat improved the impaired response of the mesenteric circulation to histamine and bradykinin caused by diabetes [39]. Aldose reductase inhibitor treatments have also been shown to prevent the depression of endothelium-dependent aortic relaxations induced by diabetes, abnormalities in contraction and improve the synthesis of prostacyclin by the aorta of diabetic rats [40-42]. In addition, in vitro studies using aortic rings incubated in 44 mM glucose demonstrated that hyperglycemia caused an aldose reductase inhibitor and myo-inositol sensitive impairment in endothelium-dependent vascular relaxation [43,44]. Recently, Keegan et al. have demonstrated that treating diabetic rats with the aldose reductase inhibitor WAY121509 completely prevented the diabetes-induced decrease in acetylcholine-induced relaxation in the corpus cavernosum vascular bed [45]. In the aorta from diabetic rats Archibald et al. and Ozyazgan et al. have demonstrated that treatment with aminoguanidine improved the diabetes-induced decrease in vascular relaxation [46,47]. This could be due to a decrease in advanced glycation end products, which have been demonstrated to quench nitric oxide activity [48]. In contrast, treating diabetic rats with aminoguanidine did not improve the diabetes-induced impairment of vascular reactivity of arterioles derived from striated muscle [49]. This suggests that improvement in vascular reactivity by aminoguanidine treatment of diabetic rats may be dependent on the vascular bed being examined. Even though many of the studies presented above focused on the influence of treatment of diabetic rats with aldose reductase inhibitors, aminoguanidine or myo-inositol on nerve function, there is little information available on the impact of these treatments on the function of blood vessels that vascularize the sciatic nerve. Since not all blood vessels are alike it is important that this issue be addressed. Thus, in the present study, using streptozotocin-induced diabetic rats of 7-8 weeks duration, we examined the effect of diabetes and its treatment with sorbinil, aminoguanidine or myo-inositol on endothelium-dependent vascular relaxation of epineurial vessels of the sciatic nerve, endoneurial blood flow and motor nerve conduction velocity.

### MATERIALS AND METHODS

Unless stated otherwise all chemicals used in these studies were obtained from Sigma Chemical Co. (St. Louis, MO). Sorbinil was a generous gift from Pfizer Pharmaceutical Co. Pentosidine standard was a kind gift from Dr. Vincent Monnier.

### *Animals*

Male Sprague-Dawley (Harlan Sprague Dawley, Indianapolis, IN) rats 8-9 weeks of age were housed in a certified animal care facility and food (Harlan Teklad, #7001, Madison, WI) and water were provided ad libitum. All institutional and NIH guidelines for use of animals were followed. Diabetes was induced by intravenously injecting streptozotocin (60 mg/kg in 0.9% NaCl, adjusted to a pH 4.0 with 0.2 M sodium citrate). Control rats were injected with vehicle alone. The rats were anesthetized with halothane before injection. Diabetes was verified 48h later by evaluating blood glucose levels with the use of glucose-oxidase reagent strips (Lifescan Inc., Milpitas, CA). Rats having blood glucose level of 300 mg/dl (16.7 mM) or greater were considered to be diabetic. At this time the diabetic rats were randomly divided into four groups. The first group received sorbinil as a dietary supplement

0.05% by weight. The second group received myo-inositol as a dietary supplement 1% by weight. The third group-received aminoguanidine delivered in the water at a dose of 1g/L. The final group represented the untreated diabetic group. The sorbinil and myo-inositol was added to a meal form of the diet that was formed into pellets for feeding purposes. Control, untreated diabetic, and diabetic rats treated with aminoguanidine were fed non-supplemented pelleted rat chow. The chow was made in the laboratory, dried in a vacuum oven and stored refrigerated until use. Water containing aminoguanidine was made fresh weekly and kept refrigerated. All studies were conducted approximately 7-8 weeks after the verification of diabetes.

On the day of the experiment blood glucose level was determined and the rats were anesthetized with Nembutal i.p. (50 mg/kg, i.p., Abbott Laboratories, North Chicago, IL). Following the determination of motor nerve conduction velocity (MNCV) and endoneurial blood flow (EBF), the abdominal aorta was isolated and occluded 1-2 cm above the branch of the common iliac artery. The rat was then sacrificed by exsanguination, and body temperature lowered with topical ice. Samples of the left sciatic nerve were then taken for determination of $Na^+/K^+$ ATPase activity and sorbitol, fructose and myo-inositol content. The lens was collected for determination of glutathione levels. Serum samples were also taken for determination of thiobarbituric acid reactive substances (TBARS) and free fatty acids and triglycerides.

### *Motor nerve conduction velocity*

MNCV was determined as previously described using a noninvasive procedure in the sciatic-posterior tibial conducting system in a temperature controlled environment [21,36].

### *Endoneurial blood flow*

Immediately after determination of MNCV, sciatic nerve endoneurial nutritive blood flow was determined as described by Cameron *et al.* [33,50]. The trachea was intubated for mechanical ventilation and a carotid cannula inserted to monitor mean arterial blood pressure. Core temperature was monitored using a rectal probe and temperature regulated between 36 and 37°C using a heating pad and radiant heat. The right sciatic nerve was carefully exposed by a small surgical incision and the surrounding skin sutured to a plastic ring. The isolated area was filled with mineral oil at 37°C to a depth of 1 cm to minimize diffusion of hydrogen gas from the nerve. The rats were then mechanically ventilated. A glass insulated platinum microelectrode (tip = 2 µm) was inserted into the sciatic nerve, proximal to the trifurcation, and polarized at 0.25 V with respect to a reference electrode inserted subcutaneously into the flank of the rat. Once the recording had stabilized, the inspired air was modified to contain 10% hydrogen gas and this gas flow continued until the hydrogen current recorded by the electrode had stabilized, indicating equilibrium of the inspired air with arterial blood. The hydrogen gas supply was then discontinued and the hydrogen clearance curve recorded until a baseline was achieved. The hydrogen clearance data was fitted to a mono- or bi-exponential curve using commercial software (Prism, GraphPad, San Diego, CA) and nutritive blood flow, (ml/min/100g), calculated using the equation described by Young [51] and vascular conductance, (ml/min/100g/mm Hg) determined by dividing nutritive blood flow by the average mean arterial blood pressure. Two recordings were made for each rat at different locations along the nerve and the final blood flow value averaged.

### *Vascular reactivity*

Videomicroscopy was used to investigate *in*

*vitro* vasodilatory responsiveness of arterioles vascularizing the region of the sciatic nerve (branches of the superior gluteal and internal pudendal arteries) as previously described [35,36]. The vessels used for these studies were generally oriented longitudinally in relation to the sciatic nerve; however, on occasion radially oriented vessels were also used. No differences were observed in acetylcholine-induced vasodilation based on the orientation of the vessel to the sciatic nerve. The arterioles used in this study should be regarded as epineurial rather than perineurial vessels. Cumulative concentration-response relationships were evaluated for acetylcholine ($10^{-8}$–$10^{-4}$ M) using vessels from control and untreated and treated diabetic rats. At the end of each acetylcholine dose response determination sodium nitroprusside ($10^{-4}$ M) was added to determine maximal vasodilation.

### *Detection of superoxide*

Hydroethidine (Molecular Probes Inc., Eugene, OR), an oxidative fluorescent dye, was used to evaluate *in situ* levels of superoxide ($O_2^-$) as described previously [35,52]. Hydroethidine is permeable to cells and in the presence of $O_2^-$ is oxidized to fluorescent ethidium bromide, where it is trapped by intercalating with DNA. This method provides sensitive detection of $O_2^-$ *in situ*. Unfixed frozen ring segments were cut into 5-µm-thick sections and placed on glass slides. Hydroethidine ($2 \times 10^{-6}$ M) was topically applied to each tissue section and cover slipped. Slides were incubated in a light protected humidified chamber at 37°C for 30 minutes. Images were obtained with a Bio-Rad MRC-1024 laser scanning confocal microscope equipped with a krypton/argon laser. Fluorescence was detected with a 585-nm long pass filter. Tissue from control and untreated and treated diabetic rats were processed and imaged in parallel. Laser settings were identical for acquisition of all images from control and diabetic specimens.

### *Sciatic nerve sorbitol, fructose and myo-inositol content and retina pentosidine level*

As a marker of sorbinil and myo-inositol treatment efficacy the level of sciatic nerve sorbitol, fructose and myo-inositol was determined. Briefly, the left sciatic nerve was removed, desheathed, and divided into two samples for determination of $Na^+/K^+$ ATPase activity and sorbitol, fructose and myo-inositol content [35]. For the determination of sorbitol, fructose and myo-inositol content the nerve sample was weighed, derivatized and analyzed by gas-liquid chromatography as previously described [21,36]. To determine aminoguanidine efficacy the pentosidine content of the retina was determined by high performance liquid chromatography coupled with fluorescence detection as described by Sell and Monnier [53].

### *$Na^+/K^+$ ATPase activity*

Total and ouabain-inhibited $Na^+/K^+$ ATPase activities were measured in crude homogenates of sciatic nerve [21,36]. Sciatic nerves were homogenized in a glass homogenizer at 4°C in 1 ml of 0.2 M sucrose, 0.02 M Tris-HCl buffer, pH 7.5. The samples were then centrifuged at 100-x g for 10 min at 4°C. An aliquot of the supernatant (50 µl) was added to two cuvettes containing 100 mM NaCl, 10 mM KCl, 2.5 mM $MgCl_2$, 2 mM ethylene glycol-bis(β-aminoethyl ether)-$N_1$-N'-tetraacetic acid (EGTA), 1 mM Tris-ATP, 1 mM 3-(cyclohexylammonium) phosphoenolpyruvate (Boehringer-Mannheim, Indianapolis, IN), 30 mM imidazole-HCl buffer (pH 7.3), 0.15 mM NADH, 50 µg lactate dehydrogenase (Boehringer-Mannheim, Indianapolis, IN), 30 µg pyruvate kinase (Boehringer-Mannheim, Indianapolis, IN) with or without 1 mM ouabain to inhibit the ouabain-sensitive $Na^+/K^+$ ATPase fraction. After a 20 min stabilization period, the oxidation of NADH was recorded over a 30 min

TABLE I Change in Body Weight and Blood Glucose Levels

| Animal | Change in Body Weight (g) | Blood glucose mg/dl |
|---|---|---|
| Control (n = 7) | 195 ± 17 | 88 ± 7 |
| Diabetic (n = 7) | 39 ± 16+ | 390 ± 22+ |
| Diabetic + sorbinil (n = 11) | 40 ± 14+ | 430 ± 22+ |
| Diabetic + myo-inositol (n = 9) | 43 ± 14+ | 425 ± 21+ |
| Diabetic + aminoguanidine (n = 11) | 24 ± 9+ | 427 ± 17+ |

Data are means ± S.E.M.
+ $p < 0.05$ vs control

TABLE II Effect of Treatment of Streptozotocin-induced Diabetic Rats with Sorbinil, myo-Inositol or Aminoguanidine on Sciatic Nerve $Na^+/K^+$ ATPase activity and Sorbitol, Fructose and myo-Inositol Levels

| Animal | $Na^+/K^+$ ATPase activity (µmol ADP/g wet weight) | Intracellular content (nmol/mg wet weight) | | |
|---|---|---|---|---|
| | | Sorbitol | Fructose | myo-Inositol |
| Control (n = 7) | 296.7 ± 22.9 | 0.2 ± 0.1 | 0.8 ± 0.1 | 10.4 ± 2.1 |
| Diabetic (n = 7) | 129.6 ± 25.2+ | 0.8 ± 0.1+ | 3.4 ± 0.2+ | 4.7 ± 0.6+ |
| Diabetic + sorbinil (n = 11) | 207.8 ± 27.5* | 0.3 ± 0.1* | 1.4 ± 0.3* | 8.9 ± 1.0* |
| Diabetic + myo-inositol (n = 9) | 186.9 ± 9.8* | 0.9 ± 0.3+ | 3.3 ± 0.4+ | 9.6 ± 0.3* |
| Diabetic + aminoguanidine (n = 11) | 111.6 ± 20.3+ | 1.1 ± 0.2+ | 4.6 ± 0.6+ | 6.1 ± 1.1+ |

Data are means ± S.E.M.
+ $p < 0.05$ vs control
* $p < 0.05$ vs diabetic period. The activity was expressed as µmol ADP/g wet weight/h. Each assay was conducted in triplicate.

### ADDITIONAL BIOLOGICAL PARAMETERS

Lens glutathione (GSH) and serum TBARS levels were determined as additional markers of oxidative stress. Lens glutathione levels were determined according to Lou et al. [54]. Lens were weighed and homogenized in 1 ml of cold 10% trichloroacetic acid and centrifuged for 15 min at 1000-x g. The supernatant (100 µl) was mixed with 0.89 ml of 1.0 M Tris, pH 8.2, and 0.02 M EDTA. Afterwards, 10 µl of dithionitrobenzene (DTNB) was added and change in absorbance measured at 412 nm. A glutathione standard curve (100-500 ng) was performed for each assay. The data were recorded as µg/mg

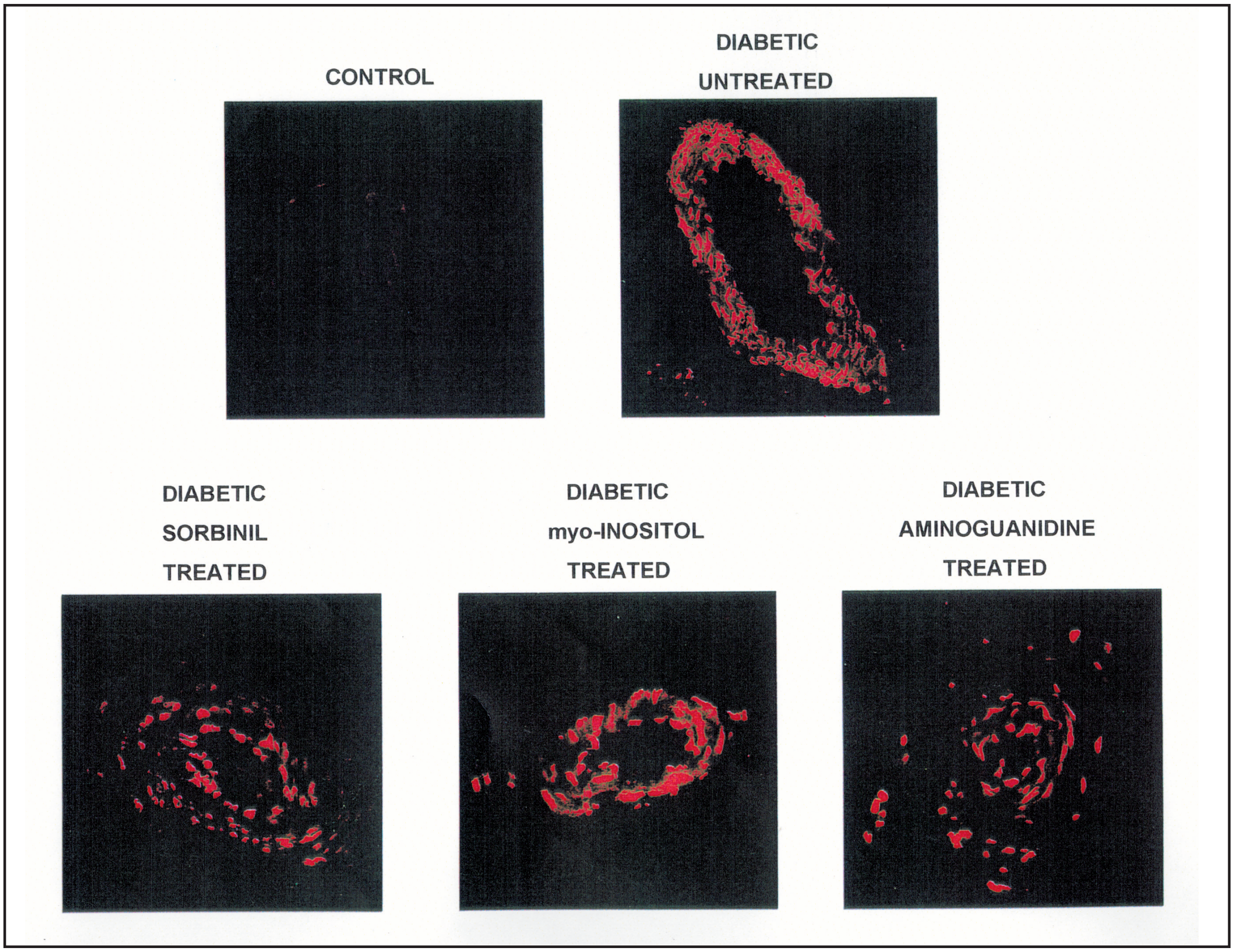

FIGURE 1
Detection of superoxide level in epineurial vessels from control, diabetic rats and diabetic rats treated with sorbinil, myo-inositol or aminoguanidine. Fluorescent photomicrographs of confocal microscopic sections of arterioles that provide circulation to the region of the sciatic nerve from the five individual groups of animals were examined on the same day. Arterioles were labeled with the oxidative dye hydroethidine as described in the Methods section. Recording of fluorescent were taken at identical laser and photomultiplier settings for both control and untreated and treated diabetic rats. Shown is a representative sample of one set of animals. This experiment was repeated four separate times on separate sets of animals with similar results.

wet weight. TBARS level in serum was determined by the method of Mihara *et al.* [55] as modified by Siman and Eriksson [56]. Briefly, 200 µl of serum was boiled in 0.75 ml of phosphoric acid (0.19 M), 0.25 ml thiobarbituric acid (0.42 mM) and 0.3 ml water for 60 min. Afterwards, the samples were precipitated with methanol/NaOH and centrifuged for 5 min. The supernatant was measured fluorometrically at excitation wavelength of 532 nm and emission wavelength of 553 nm. Standards were prepared by the acid hydrolysis of 1,1,3,3-tetraethoxypropane. The data was reported as µg/ml serum. Serum free fatty acid and triglyceride levels were determined using commercial kits from Roche Diagnostics, Mannheim, Germany and Sigma Chemical Co., St. Louis, MO, respectively.

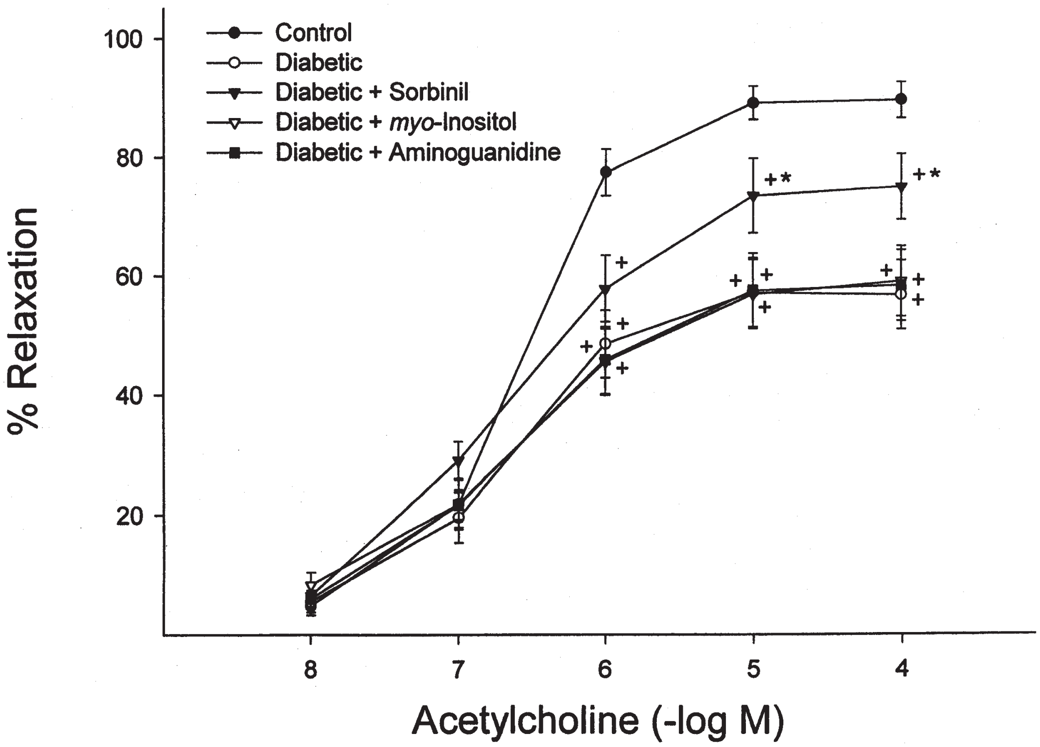

FIGURE 2
Determination of the effect of treatment of diabetic rats with sorbinil, myo-inositol or aminoguanidine on acetylcholine-mediated vascular relaxation in arterioles that provide circulation to the region of the sciatic nerve. Pressurized arterioles were constricted with U46619 (30-50%) and incremental doses of acetylcholine were added to the bathing solution while recording steady state vessel diameter. The number of experimental animals used in these studies was the same as noted in Table I. The + denotes that the response to acetylcholine was significantly attenuated in the diabetic rats compared to control rats. The * denotes that the response to acetylcholine was significantly different compared to the untreated diabetic rats.

### *Data Analysis*

The results are presented as mean ± SE. Comparisons between the groups for MNCV, EBF, sciatic nerve $Na^+/K^+$ ATPase activity, sciatic nerve sorbitol, fructose and myo-inositol content, serum TBARS, serum free fatty acid and triglyceride and lens glutathione levels were conducted using independent unpaired Student's t tests. Dose response curves for acetylcholine-induced relaxation were compared using a two way repeated measures analysis of variance with autoregressive covariance structure using proc mixed program of SAS [35,36]. Whenever significant interactions were noted specific treatment-dose-effects were analyzed using a Bonferroni adjustment. A p value of less 0.05 was considered significant. All computations were performed using SAS for Windows version 6.12.

### RESULTS

### *Body weight and plasma glucose levels*

Data in Table I show that streptozotocin-induced diabetic rats treated or untreated gained less weight than age-matched control rats over the 7-8 week period of this study. At the time of experimentation plasma glucose levels were increased 4-fold in treated or untreat-

TABLE III Effect of Treatment of Streptozotocin-induced Diabetic Rats with Sorbinil, myo-Inositol or Aminoguanidine on Serum Free Fatty Acid, Triglyceride and Thiobarbituric Acid Reactive Substances (TBARS) and Lens Glutathione Levels

| Animal | Serum | | | Lens |
|---|---|---|---|---|
| | Free Fatty Acid (mmol/l) | Triglyceride (mg/dl) | TBARS (μg/ml) | Glutathione (μg/mg wet wt) |
| Control (n = 7) | 0.10 ± 0.01 | 69 ± 7 | 8.2 ± 1.2 | 1.46 ± 0.09 |
| Diabetic (n = 7) | 0.70 ± 0.06$^{+}$ | 543 ± 172$^{+}$ | 20.2 ± 2.3$^{+}$ | 0.32 ± 0.08$^{+}$ |
| Diabetic + sorbinil (n = 11) | 0.68 ± 0.05$^{+}$ | 448 ± 77$^{+}$ | 15.3 ± 1.5$^{+}$ | 1.30 ± 0.11$^{*}$ |
| Diabetic + myo-inositol (n = 9) | 0.67 ± 0.05$^{+}$ | 473 ± 125$^{+}$ | 14.3 ± 1.8$^{+*}$ | 0.25 ± 0.04$^{+}$ |
| Diabetic + aminoguanidine (n = 11) | 1.13 ± 0.28$^{+}$ | 353 ± 47$^{+}$ | 16.2 ± 1.2$^{+}$ | 0.28 ± 0.05$^{+}$ |

Data are means ± S.E.M.
$^{+}$ $p < 0.05$ vs control
$^{*}$ $p < 0.05$ vs diabetic ed diabetic rats compared to control rats. Treating diabetic rats with sorbinil, myo-inositol or aminoguanidine had no significant effect on weight gain or blood glucose level compared to untreated diabetic rats.

### *Sciatic nerve $Na^+/K^+$ ATPase activity and sorbitol, fructose and myo-inositol content*

Data in Table II demonstrate that diabetes causes a significant decrease in sciatic nerve $Na^+/K^+$ ATPase activity. Treating diabetic rats with sorbinil or myo-inositol significantly improved sciatic nerve $Na^+/K^+$ ATPase activity. In contrast, treating diabetic rats with aminoguanidine did not prevent the diabetes-induce decrease in $Na^+/K^+$ ATPase activity. Data in Table II also demonstrate that diabetes causes a significant increase in the sorbitol and fructose content in the sciatic nerve and a decrease in the myo-inositol content. Treating diabetic rats with sorbinil corrected this defect, whereas treating diabetic rats with myo-inositol only corrected the myo-inositol content in the sciatic nerve. Treating diabetic rats with aminoguanidine does not correct the changes in sorbitol, fructose or myo-inositol levels in the sciatic nerve. The latter data demonstrate the efficacy of the sorbinil and myo-inositol treatment of the diabetic rats. Retina pentosidine level was increased approximately 4-fold by diabetes and was totally prevented by treatment with aminoguanidine, thereby demonstrating the efficacy of the aminoguanidine treatment (data not shown).

### *Serum triglyceride and free fatty acid levels*

Data in Table III demonstrate that diabetes causes a significant increase in serum free fatty acid and triglyceride levels, which was not affected by treatment with sorbinil, myo-inositol or aminoguanidine.

### *Evaluation of oxidative stress*

In order to assess the effect of diabetes and its treatment with sorbinil, myo-inositol or aminoguanidine on oxidative stress, we examined three different markers of oxidative stress in several different tissues. By examining multi-

TABLE IV Effect of Treatment of Streptozotocin-induced Diabetic Rats with Sorbinil, myo-Inositol or Aminoguanidine on Endoneurial Blood Flow and Motor Nerve Conduction Velocity (MNCV)

| Animal | Endoneurial Blood Flow | | MNCV (m/sec) |
|---|---|---|---|
| | Nutritive (ml/min/100 g) | Conductance (ml/min/100 g/mm Hg) | |
| Control (n = 7) | 19.2 ± 2.6 | 0.15 ± 0.02 | 60.2 ± 3.0 |
| Diabetic (n = 7) | 6.8 ± 1.2$^{+}$ | 0.06 ± 0.01$^{+}$ | 41.3 ± 1.3$^{+}$ |
| Diabetic + sorbinil (n = 11) | 16.3 ± 4.1$^{*}$ | 0.14 ± 0.04$^{*}$ | 49.7 ± 1.6$^{*+}$ |
| Diabetic + myo-inositol (n = 9) | 15.8 ± 2.1$^{*}$ | 0.13 ± 0.02$^{*}$ | 52.0 ± 2.4$^{*+}$ |
| Diabetic + aminoguanidine (n = 11) | 18.1 ± 2.9$^{*}$ | 0.16 ± 0.03$^{*}$ | 51.0 ± 1.6$^{*+}$ |

Data are means ± S.E.M.
$^{+}$ $p < 0.05$ vs control
$^{*}$ $p < 0.05$ vs diabetic ple markers of oxidative stress we hoped to get a better understanding of the oxidative stress status of the diabetic rats and the effect of treatment with sorbinil, myo-inositol or aminoguanidine. Data in Table III demonstrate that diabetes causes a significant increase in thiobarbituric acid reactive substances (TBARS) in serum. Treating diabetic rats with sorbinil, myo-inositol or aminoguanidine did not affect the increase in serum TBARS level caused by diabetes. Data in Table III also demonstrate that lens glutathione level was significantly decreased in streptozotocin-induced diabetic rats compared to control rats. Treating diabetic rats with myo-inositol or aminoguanidine did not improve the decrease in lens glutathione level induced by diabetes. In contrast, treating diabetic rats with sorbinil significantly improved lens glutathione level.

We have previously reported that diabetes causes an increase in superoxide level in arterioles that provide circulation to the region of the sciatic nerve [35]. The increase in superoxide level was observed in endothelial cells as well as in the smooth muscle and adventitial cells. In these studies we sought to determine whether treating diabetic rats with sorbinil, myo-inositol or aminoguanidine prevented the increase in superoxide level observed in vascular tissue. Data in Figure 1 demonstrate that treating streptozotocin-induced diabetic rats with sorbinil, myo-inositol or aminoguanidine did not prevent the diabetes-induced increase in the level of superoxide in epineurial vessels as measured by hydroethidine fluorescence compared to paired analysis of untreated diabetic rats. The apparent small improvement in superoxide level in epineurial vessels from diabetic rats treated with sorbinil or aminoguanidine was vividly less than observed following treatment with antioxidants [52,57].

### *Endoneurial blood flow and motor nerve conduction velocity*

As previously reported, diabetes causes a reduction in EBF and slowing of MNCV in the sciatic nerve conducting system [35,36]. Data in Table IV demonstrate that treating diabetic rats with sorbinil, myo-inositol or aminoguanidine prevents the decrease in EBF and MNCV compared to untreated diabetic rats.

### *Arteriolar vascular reactivity*

Stimulated changes in vascular diameter of arterioles that provide circulation to the region of the sciatic nerve were measured *in vitro* by application of acetylcholine (endothelium-dependent) as previously described [35,36]. Baseline diameter of vessels from control and diabetic rats (untreated or treated) was similar and the vessels were constricted to a similar degree with U46619 (10-100 nM). As previously reported, and as demonstrated in Figure 2, diabetes causes a significant decrease in acetylcholine mediated vascular relaxation in arterioles that provide circulation to the region of the sciatic nerve. Treating diabetic rats with sorbinil significantly improved by about 50% the diabetes-induced impairment in acetylcholine mediated vascular relaxation. In contrast, treating diabetic rats with myo-inositol or aminoguanidine did not improve acetylcholine mediated vascular relaxation. Maximal vascular relaxation induced by $10^{-4}$ M sodium nitroprusside (endothelium-independent) in these vessels was significantly reduced by diabetes ($99.5 \pm 2.1$ vs $76.2 \pm 5.7$ for control and diabetic rats, respectively, $p < 0.05$). Treating diabetic rats with sorbinil, myo-inositol or aminoguanidine did not significantly improve maximal vascular relaxation induced by $10^{-4}$ M sodium nitroprusside ($86.3 \pm 4.6$, $75.4 \pm 3.9$ and $76.3 \pm 4.9$, respectively, $p < 0.05$ compared to control).

### DISCUSSION

Diabetic neuropathy is a heterogeneous disease with a widely varying pathology and multiple etiologies including metabolic, vascular, autoimmune, oxidative stress, and neurohormonal growth-factor deficiency components [58]. Among the metabolic factors that potentially contribute to the development of diabetic neuropathy the polyol pathway and nonenzymatic glycation have been extensively studied [59]. Based on these studies pharmacological compounds, like aldose reductase inhibitors and anti-glycation agents, have been developed for the treatment of diabetic neuropathy [59]. In diabetic animal models these agents have been shown to improve nerve function and endoneurial blood flow [3-12,24-28]. However, studies on the effect these agents may have on vascular function of vessels that provide circulation to nerves have been limited. We have previously shown that endothelial-dependent vascular relaxation of arterioles that provide circulation to the region of the sciatic nerve is impaired by diabetes [35,36]. In the present study we sought to determine the effect treatment of diabetic rats with sorbinil, an aldose reductase inhibitor, aminoguanidine, an inhibitor of nonenzymatic glycation, or myo-inositol supplementation has on EBF, MNCV as well as vascular relaxation of arterioles that provide circulation to the region of the sciatic nerve.

As has been previously reported, we demonstrate that treating streptozotocin-induced diabetic rats with sorbinil or aminoguanidine significantly improves the reduction in EBF and slowing of MNCV [3-12,24-28]. In sorbinil treated diabetic rats EBF and MNCV remained suppressed by 15 and 18%, respectively compared to controls, whereas in aminoguanidine treated rats EBF and MNCV, compared to controls, was decreased by 6 and 16%, respectively. This indicates that aminoguanidine treatment was more effective than sorbinil in improving EBF. This could be interpreted as meaning that pathways inhibited by aminoguanidine in diabetic rats such as increased nonenzymatic glycation could have a greater impact on reducing EBF than increased aldose reductase activity. However, the efficacy of these treatments could also be different. The dose of sorbinil used in our studies did not completely prevent the diabetes-induced increase in sorbitol and fructose levels in the sciatic nerve suggesting that we did not achieve total inhibition of aldose reductase activity in our studies, which could have an impact on the differences observed in the efficacy of these treatments. Dietary myo-inositol supplementation also improved the slowing of MNCV as previously reported as well as EBF [18-21]. The latter result was unexpected since Cameron et al. [9] has reported that aldose reductase inhibitor treatment of diabetic rats improves the reduction in EBF and MNCV independent of nerve myo-inositol levels. In contrast, Stevens et al. [60] has reported that myo-inositol supplementation improves EBF in diabetic rats. Therefore, the role of myo-inositol deficiency in the development of diabetic neuropathy remains controversial [61]. Clinically, myo-inositol supplementation has proved disappointing and no trials have shown any improvement in nerve function [62].

Treatment of diabetic rats with aminoguanidine or myo-inositol did not improve the diabetes-induced impairment in acetylcholine-mediated vascular relaxation in arterioles that provide circulation to the region of the sciatic nerve, suggesting that neither treatment is efficacious in preventing diabetic vascular disease in this vascular bed as it relates to diabetic neuropathy. In contrast, our studies demonstrated that sorbinil treatment of diabetic rats significantly improved vascular reactivity in epineurial vessels. This agrees with previous studies that demonstrated that treating diabetic rats with an aldose reductase inhibitor improved relaxation in the aorta, corpus cavernosum and mesenteric vascular beds [42,45]. The observation that treatment of diabetic rats with aminoguanidine or myo-inositol corrects EBF without improving vascular reactivity of epineurial vessels is intriguing. This would suggest that regulation of EBF is not completely dependent upon vascular relaxation of epineurial vessels and that reduced EBF can be improved in diabetic animal models independent of correction of vascular function of epineurial vessels. The mechanism responsible for this phenomenon is unknown but could include differences of the effect of hyperglycemia/diabetes on the vascular function of epineurial vessels compared to endoneurial vessels. One possibility could be the effect of hyperglycemia/diabetes on the vascular smooth muscles of epineurial vessels and the anatomical differences between epineurial and endoneurial vessels. In this regard, it has been reported that epineurial and perineurial vessels are less significantly diseased than endoneurial vessels in diabetes [63].

In agreement with our studies, Crijns et al. [49] found that treating diabetic rats with aminoguanidine had no beneficial effect on the reduction of endothelium-dependent vasodilation of arterioles derived from striated muscle. This contrasts with previous studies that demonstrated that aminoguanidine treatment of diabetic rats improved vasodilation in the aorta [46,47]. In addition, Cartledge et al. [64] have demonstrated that the formation of advanced glycation endproducts is probably responsible for the impairment of endothelial-dependent penile smooth muscle relaxation seen in diabetes. This suggests that the mechanisms responsible for vascular dysfunction in diabetes may be partially dependent on the vascular bed. In contrast, to the lack of a beneficial effect of aminoguanidine or myo-inositol treatment of diabetic rats on vascular function observed in our studies of epineurial vessels, treating diabetic rats with sorbinil improved acetylcholine-mediated vascular relaxation by about 50%. Other studies have demonstrated that diabetes-induced impairment of vascular reactivity in the corpus cavernosum, mesentery, and aorta are corrected by treatment with aldose reductase inhibitors [39,40,42,45]. Interestingly, sorbinil treatment restored lens glutathione levels to normal and improved the reduction in sciatic nerve sorbitol, fructose and myo-inositol content and $Na^+/K^+$ ATPase activity. The improvement of glutathione levels by sorbinil treatment could be of significance since Bravenboer et al. [65] have demonstrated that glutathione, a free radical scavenger, is partially effective in preventing diabetic neuropathy in streptozotocin-induced diabetic rats. In contrast, sorbinil treatment of diabetic rats did not improve other markers of oxidative stress such as serum TBARS level or epineurial vessel superoxide level. As mentioned above, the sorbinil dosage used in our studies did not totally correct the diabetes-induced increase in sorbitol levels in the sciatic nerve or the decrease in myo-inositol. In future studies it would be important to determine whether complete inhibition of aldose reductase with either a higher concentration of sorbinil or use of a more potent aldose reductase inhibitor may further improve vascular function and perhaps other markers of oxidative stress. Treating diabetic rats with aminoguanidine did not improve any of the sciatic nerve metabolic abnormalities or markers of oxidative stress. The latter finding is surprisingly since many of the effects of increased nonenzymatic glycation and the formation of advanced glycation end products has been attributed to increasing oxidative stress [31,32]. However, much of this could be due to our lack of understanding the source of superoxide production in diabetes and the effects of these treatments. Treating diabetic rats with myo-inositol did not improve any of the markers for oxidative stress but did improve sciatic nerve myo-inositol levels and $Na^+/K^+$ ATPase activity.

Previously we have demonstrated that treating diabetic rats with two different antioxidants, α-lipoic acid or M40403, prevented the reduction in EBF, MNCV and impairment of vascular relaxation of epineurial vessels [52,57]. Treatment with these antioxidants prevented the generation of superoxide and peroxynitrite in epineurial vessels and the aorta and the increase in serum TBARS but did not correct the metabolic derangements of the sciatic nerve. Form these studies we concluded that diabetes-induced oxidative stress and the generation of superoxide and perhaps peroxynitrite may be partially responsible for the development of diabetic vascular and neural complications. The present study demonstrated that treatment of streptozotocin-induced diabetic rats with sorbinil prevented the metabolic derangements in the sciatic nerve as well as the reduction of glutathione in the lens. These defects were not uniformly corrected by treatment with antioxidants [52,57]. Thus, treatment protocols utilizing the combination of antioxidants and aldose reductase inhibitors may be an effective approach in preventing diabetic vascular and neural disease.

### *Acknowledgments*

This work was supported by a National Institute of Diabetes and Digestive and Kidney Diseases Grant DK-25295, by a grant from the National Institute of Diabetes and Digestive and Kidney Diseases DK-58005, by a Diabetes Center Grant from the Veterans Affairs and International Juvenile Diabetes Foundation, and by a research grant from the American Diabetes Association.

### REFERENCES

1. Tomlinson, D. (1998) Future prevention and treatment of diabetic neuropathy, *Diabetes & Metabolism,* **24,** 79-83.
2. Akbari, C.M. and LoGerfo, F.W. (1999) Diabetes and peripheral vascular disease, *J. Vasc. Surg.,* **30**, 373-384.
3. Finegold, D., Lattimer, S.A., Nolle, S., Bernstein, M. and Greene, D.A. (1983) Polyol pathway activity and myo-inositol metabolism, *Diabetes,* **32**, 988-992.
4. Mayer, J.H. and Tomlinson, D.R. (1983) Prevention of defects of axonal transport and nerve conduction velocity by oral administration of myo-inositol or an aldose reductase inhibitor in streptozotocin-diabetic rats, *Diabetologia,* **25**, 433-438.
5. Tomlinson, D.R., Moriaity, R.J. and Heidi Mayer, J. (1984) Prevention and reversal of defective axonal transport and motor nerve conduction velocity in rats with experimental diabetes by treatment with the aldose reductase inhibitor sorbinil, *Diabetes,* **33,** 470-476.

6. Greene, D.A. and Lattimer, S.A. (1984) Action of sorbinil in diabetic peripheral nerve, *Diabetes,* **33**, 712-716.
7. Kikkawa, R., Hatanaka, I., Yasuda, H., Kobayashi, N. and Shigeta, Y. (1984) Prevention of peripheral nerve dysfunction by an aldose reductase inhibitor in streptozotocin-diabetic rats, *Metabolism,* **33,** 212-214.
8. Sima, A.A.F., Prashar, A., Zhang, W-X., Chakrabarti, S. and Greene, D.A. (1990) Preventive effect of long-term aldose reductase inhibition (Ponalrestat) on nerve conduction and sural nerve structure in the spontaneously diabetic bio-breeding rat, *J. Clin. Invest.,* **85,** 1410-1420.
9. Cameron, N.E., Cotter, M.A., Dines, K.C., Maxfield, E.K., Carey, F. and Mirrlees, D.J. (1994) Aldose reductase inhibition, nerve perfusion, oxygenation and function in streptozotocin-diabetic rats: dose-response considerations and independence from a myo-inositol mechanism, *Diabetologia,* **37,** 651-663.
10. Hirata, Y., Fujimori, S. and Okada, K. (1988) Effect of a new aldose reductase inhibitor, 8'-Chloro-2',3'-Dihydrospiro[Pyrrolidine-3,6'(5'H)-Pyrrolol[1,2,3-de][1,4]Benzoxamine]-2,5,5'-Trione (ADN-138), on delayed motor nerve conduction velocity in streptozotocin-diabetic rats, *Metabolism,* **37,** 159-163.
11. Cameron, N.E. and Cotter, M.A. (1992) Dissociation between biochemical and functional effects of the aldose reductase inhibitor, ponalrestat, on peripheral nerve in diabetic rats, *Br. J. Pharmacol.,* **107,** 939-944.
12. Yoshida, T., Nishioka, H., Yoshioka, K., Nakano, K., Kondo, M. and Terashima, H. (1987) Effect of aldose reductase inhibitor ONO 2235 on reduced sympathetic nervous system nerve disorders in STZ-induced diabetic rats, *Diabetes,* **36,** 6-13.
13. Tomlinson, D.R., Stevens, E.J. and Diemel, L.T. (1994) Aldose reductase inhibitors and their potential for the treatment of diabetic complications, *TiPS,* **15,** 293-297.
14. Hotta, N. (1995) New approaches for treatment in diabetes: aldose reductase inhibitors, *Biomed. & Pharmacother.,* **5,** 232-243.
15. Yabe-Nishimura, C. (1998) Aldose reductase in glucose toxicity: a potential target for the prevention of diabetic complications, ***Pharmacological Rev.,*** **50,** 21-33.
16. Crabbe, M.J.C. and Goode, D. (1998) Aldose reductase: a window to the treatment of diabetic complications?, *Prog. Retinal Eye Res.,* **17,** 313-383.
17. Sugimoto, K., Murakawa, Y. and Sima, A.A.F. (2000) Diabetic neuropathy- a continuing enigma, *Diabetes/Metabolism Res. Rev.,* **16,** 408-433.
18. Greene, D.A., De Jesus, P.V. Jr. and Winegrad, A.I. (1975) Effects of insulin and dietary myoinositol on impaired peripheral motor nerve conduction velocity in acute streptozotocin diabetes, J. Clin. Invest., **55,** 1326-1336.
19. Greene, D.A., Lewis, R.A., Lattimer, S.A. and Brown, M.J. (1982) Selective effects of myo-inositol administration on sciatic and tibial motor nerve conduction parameters in the streptozotocin-diabetic rat, *Diabetes,* **31,** 573-578.
20. Greene, D.A., Chakrabarti, S., Lattimer, S.A. and Sima, A.A.F. Role of sorbitol accumulation and myo-inositol depletion in paranodal swelling of large myelinated nerve fibers in the insulin-deficient spontaneously diabetic bio-breeding rat, *J. Clin. Invest.,* **79,** 1479-1485.
21. Yorek, M.A., Wiese, T.J. Davidson, E.P., Dunlap, J.A. Stefani, M.R., Conner, C.E., Lattimer, S.A., Kamijo, M. Greene, D.A. and Sima, A.A.F. (1993) Reduced motor nerve conduction velocity and $Na^+$-$K^+$ -ATPase activity in rats maintained on L-fucose diet, *Diabetes,* **42**, 1401-1406.
22. Winegrad, A.I. (1987) Does a common mechanism induce the diverse complications of diabetes?, *Diabetes,* **36,** 396-406.
23. Nilsson, B.-O. (1999) biological effects of aminoguanidine: an update, *Inflamm. Res.,* **48,** 509-515.
24. Kihara, M., Schmelzer, J.D., Poduslo, J.F., Curran, G.L., Nickander, K.K. and Low, P.A. (1991) Aminoguanidine effects on nerve blood flow, vascular permeability, electrophysiology, and oxygen free radicals, *Proc. Natl. Acad. Sci.,* **88,** 6107-6111.
25. Cameron, N.E., Cotter, M.A., Dines, K. and Love, A. (1992) Effects of aminoguanidine on peripheral nerve function and polyol pathway metabolites in streptozotocin-diabetic rats, *Diabetologia,* **35,** 946-950.
26. Miyauchi, Y., Shikama, H., Takasu, T., Okamiya, H., Umeda, M., Hirasaki, E., Ohhata, I., Nakayama, H. and Nakagawa, S. (1996) Slowing of peripheral motor nerve conduction was ameliorated by aminoguanidine in streptozotocin-induced diabetic rats, *European J. Endo.,* **134,** 467-473.
27. Monnier, V.M. (1996) Aminoguanidine and diabetic neuropathy, *European J. Endo.,* **134,** 398-400.
28. Dewhurst, M., Omawari, N. and Tomlinson, D.R. (1997) Aminoguanidine-effects on endoneurial vasoactive nitric oxide and on motor nerve conduction velocity in control and streptozotocin-diabetic rats, *Br. J. Pharm.,* **120,** 593-598.
29. Vlassara, H., Brownlee, M. and Cerami, A. (1983) Excessive nonenzymatic glycosylation of peripheral and central nervous system myelin components in diabetic rats, *Diabetes,* **32,** 670-674.
30. Sugimoto, K., Nishizawa, Y., Horiuchi, S. and Yagihashi, S. (1997) Localization in human diabetic peripheral nerve of Ne-carboxymethyllysine-protein adducts, an advanced glycation endproduct, *Diabetologia,* **40,** 1380-1387.
31. Vlassara, H., Brownlee, M. and Cerami, A. (1986) Nonenzymatic glycosylation: role in the pathogenesis of diabetic complications, *Clin. Chem.,* **32,** B37-B41.
32. Brownlee, M., Cerami, A. and Vlassara, H. (1988) Advanced glycosylation end products in tissue and the biochemical basis of diabetic complications, *New Engl. J. Med.,* **318,** 1315-1321.
33. Cameron, N.E., Cotter, M.A. and Low, P.A. (1991) Nerve blood flow in early experimental diabetes in rats: relation to conduction deficits, *Am. J. Physiol.,* **261**, E1-E8.
34. Wright, R.A. and Nukada, H. (1994) Vascular and metabolic factors in the pathogenesis of experimental diabetic neuropathy in mature rats, *Brain,* **117**, 1395-1407.

35. Coppey, L.J., Davidson, E.P., Dunlap, J.A., Lund, D.D. and Yorek, M.A. (2000) Slowing of motor nerve conduction velocity in streptozotocin-induced diabetic rats is preceded by impaired vasodilation in arterioles that overlie the sciatic nerve, *Int. J. Exp. Diab. Res.,* **1**, 131-143.

36. Terata, K., Coppey, L.J., Davidson, E.P., Dunlap, J.A., Gutterman, D.D. and Yorek, M.A. (1999) Acetylcholine-induced arteriolar dilation is reduced in streptozotocin-induced diabetic rats with motor nerve dysfunction, *Br. J. Pharm.,* **128**, 837-843.

37. Kihara, M. and Low, P.A. (1995) Impaired vasoreactivity to nitric oxide in experimental diabetic neuropathy, *Exp. Neurology,* **132,** 180-185.

38. Maxfield, E.K., Cameron, N.E. and Cotter, M.A. (1997) Effects of diabetes on reactivity of sciatic vasa nervorum in rats, *J. Diabetes & Comp.,* **11,** 47-55.

39. Fortes, Z.B., Becker, C., Oliveira, M.A. and Scivoletto, R. (1996) Influence of aldose reductase inhibition on the microvascular reactivity in experimental diabetes, *Gen. Pharmac.,* **27,** 917-921.

40. Otter, D.J. and Chess-Williams, R. (1994) The effects of aldose reductase inhibition with ponalrestat on changes in vascular function in streptozotocin diabetes rats, *Br. J Pharm.,* **113,** 576-580.

41. Wakasugi, M., Noguchi, T., Inoue, M., Tawata, M., Shindo, H. and Onaya, T. (1991) Effects of aldose reductase inhibitors on prostacyclin ($PGI_2$) synthesis by aortic rings from rats with streptozotocin-induced diabetes, *Prostaglandins, Leukotrienes and Essential Fatty Acids,* **44**, 233-236.

42. Cameron, N.E. and Cotter, M.A. (1992) Impaired contraction and relaxation in aorta from streptozotocin-diabetic rats: role of polyol pathway, *Diabetologia,* **35,** 1011-1019.

43. Tesfamariam, B., Brown, M.L. and Cohen, R.A. (1992) Aldose reductase and myo-inositol in endothelial cell dysfunction caused by elevated glucose, *J. Pharm. Exp. Therap.,* **263,** 153-157.

44. Tesfamariam, B., Palacino, J.J., Weisbrod, R.M. and Cohen, R.A. (1993) Aldose reductase inhibition restores endothelial cell function in diabetic rabbit aorta, *J. Cardiovasc. Pharm.,* **21,** 205-211.

45. Keegan, A., Jack, A.M., Cotter, M.A. and Cameron, N.E. (2000) Effects of aldose reductase inhibition on responses of the corpus cavernosum and mesenteric vascular bed of diabetic rats, *J. Cardiovasc. Pharm.,* **35**, 606-613.

46. Archibald, V., Cotter, M.A., Keegan, A. and Cameron, N.E. (1996) Contraction and relaxation of aortas from diabetic rats: effects of chronic anti-oxidant and aminoguanidine treatments, *Naunyn-Schmiedebergs Arch. Pharm.,* **353,** 584-591.

47. Ozyazgan, S., Unluccerci, Y., Bekpinar, S. and Akkan, A.G. (2000) Impaired relaxation in aorta from streptozotocin-diabetic rats: effect of aminoguanidine (AMNG) treatment, *Int. J. Exp. Diabetes Res.,* **1,** 145-153.

48. Bucala, R. Tracey, K.J. and Cerami, A. (1991) Advanced glycosylation products quench oxide and mediate defective endothelium-dependent vasodilatation in experimental diabetes, *J. Clin. Invest.,* **87,** 432-438.

49. Crijns, F.R.L., Struijker Boudier, H.A. and Wolffenbuttel, B.H.R. (1998) Arteriolar reactivity in conscious diabetic rats: influence of aminoguanidine treatment, *Diabetes,* **47,** 918-923.

50. Cameron, N.E., Cotter, M.A., Basso, M. and Hohman, T.C. (1997) Comparison of the effects of inhibitors of aldose reductase and sorbitol dehydrogenase on neurovascular function, nerve conduction and tissue polyol pathway metabolites in streptozotocin-diabetic rats, *Diabetologia,* **40**, 271-281.

51. Young, W. (1980) $H_2$ clearance measurement of blood flow: a review of technique and polarographic principles, *Stroke,* **11**, 552-564.

52. Coppey, L.J., Gellett, J.S., Davidson, E.P., Dunlap, J.A., Lund, D.D. and Yorek, M.A. (2001) Effect of antioxidant treatment of streptozotocin-induced diabetic rats on endoneurial blood flow, motor nerve conduction velocity and vascular reactivity of epineurial arterioles of the sciatic nerve, *Diabetes,* **50**, 1927-1937.

53. Sell, D.R. and Monnier, V.M. (1989) Structure elucidation of a senescence cross-link from human extracellular matrix, *J. Biol. Chem.,* **264**, 21597-21602.

54. Lou, M.F., Dickerson, J.E. Jr, Garadi, R. and York, B.M. Jr. (1988) Glutathione depletion in the lens of galactosemic and diabetic rats, *Exp. Eye Res.,* **46**, 517-530.

55. Mihara, M., Uchiyama, M. and Fukuzama, K. (1980) Thiobarbituric acid value of fresh homogenate of rat as a parameter of lipid peroxidation in aging, CC14 intoxication, and vitamin E deficiency, *Biochem. Med.,* **23**, 302-311.

56. Siman, C.M. and Eriksson, U.J. (1997) Vitamin C supplementation of the maternal diet reduces the rate of malformation in the offspring of diabetic rats, *Diabetologia,* **40**, 1416-1424.

57. Coppey, L.J., Gellett, J.S., Davidson, E.P., Dunlap, J.A., Lund, D.D., Salvemini, D and Yorek, M.A. (2001) Effect of M40403 treatment of diabetic rats on endoneurial blood flow, motor nerve conduction velocity and vascular function of epineurial arterioles of the sciatic nerve, *Br. J. Pharm.,* **134,** 21-29.

58. Vinik, A.I., Park, T.S., Stansberry, K.B. and Pittenger, G.L. (2000) Diabetic neuropathies, *Diabetologia,* **43**, 957-973.

59. Yagihasshi, S. (1995) Pathology and pathogenetic mechanisms of diabetic neuropathy, *Diabetes/Metabolism Rev.,* **11**, 193-225.

60. Stevens, M., Van Huysen, C., Beyer, L., Thomas, T. and Yorek, M.A. (1996) Amelioration of nerve blood flow deficits by myo-inositol in streptozotocin-diabetic rats, *Diabetes,* **45**, 210A.

61. Loy, A., Lurie, K.G., Ghosh, A., Wilson, J.M., MacGregor, L.C. and Matschinsky, F.M. (1990) Diabetes and the myo-inositol paradox, *Diabetes,* **39**, 1305-1312.

62. Tomlinson, D.R. (1992) The pharmacology of diabetic neuropathy, *Diabetes/Metabolism Rev.,* **8**, 67-84.

63. Ward, J.D. (1995) Biochemical and vascular factors in the pathogenesis of diabetic neuropathy, *Clin. Invest. Med.,* **18**, 267-274.

64. Cartledge, J.J., Eardley, I. and Morrison, J.F.B. (2001) Advanced glycation end-products are responsible for the impairment of corpus cavernosal smooth muscle relaxation seen in diabetes, *BJU International,* **87**, 402-407.

65. Bravenboer, B., Kappelle, A.C., Hamers, F.P.T., van Buren, T., Erkelens, D.W. and Gispen, W.H. (1992) Potential use of glutathione for the prevention and treatment of diabetic neuropathy in the streptozotocin-induced diabetic rat, *Diabetologia,* **35**, 813-817.

